# 

**Published:** 1891-12-12

**Authors:** 


**Notices of Births, Deaths, and Marriages,** are inserted in this column at a charge of 2s. 6d. for an announcement not exceeding four lines.

### NOTICE TO CORRESPONDENTS.

All MS., Letters, Books for Review, and other matters intended for the Editor should be addressed The Editor, The Lodge, Porchester Square, London, W.

The Editor cannot undertake to return rejected M.S., even when accompanied by stamped directed envelope.

**Notice to Advertisers and Subscribers.**

All Advertisements, Orders for Copies of The Hospital, and Business Communications should be addressed to The Publisher, *not to the Editor*, The Hospital, 140, Strand, W.C.

The Cost of Subscription, post free, is as follows:—Per week, 1½d.; per quarter, 1s. 8d.; per half-year, 3s. 3d.; per annum, 6s. 6d. Foreign:—Per annum, 8s. 8d.; payable, in all cases, in advance.

**"Burdett's Hospital Annual," 1891—1892.**

Third year of publication. 600 pp. Boards, gilt lettering. A most complete guide to British and Colonial Hospitals, Dispensaries, Nursing and Convalescent Institutions, and Asylums. Price 3s. 6d. Published by The Hospital (Limited), 140, Strand, W.C.

**NOTICE.—The Index for Vol. X. (ending September, 1891), is on sale at the Office of this Journal, price 2½d., post free.**

### General Charities.

[This Column is devoted to an account of work of charitable institutions other than Medical Charities. The Editor will be glad to receive reports or news of work at 140, Strand. *Philanthropy* should be plainly written on the envelope.]

The Duchess of Teck has become a patroness of the Church Society for Providing Homes for Waifs and Strays.

Donations to the amount of nearly £700 were given in response to Mr. Lockwood's appeal at the recent dinner on behalf of the Royal General Theatrical Fund.

General Sir Robert Phayre, K.C.B., and Rev. Canon Fleming, B.D., Chaplain to the Queen, have become the Vice-Presidents of Dr. Barnardo's Homes, now containing 4,200 rescued waif children.

The recent Naval Exhibition promised from the first to be a great success, but probably the most sanguine believers in the prosperity of the undertaking were not prepared to hear that the Executive have found themselves able to set aside from the profits, as it is stated, a sum of nearly £70,000 for the benefit of a **ROYAL NAVAL EXHIBITION FUND**, which is to be of a permanent character, for the support of the widows and orphans of sailors and marines who may lose their lives in Her Majesty's service.

Manchester and Salford have set an excellent example to other wealthy centres in this country by starting a permanent Lifeboat Saturday Fund. If this example, as is to be hoped, is followed in other cities, not only will the financial difficulty of the Royal Lifeboat Society be a thing of the past, but the scope of its work would be enlarged in many useful ways which are at present unattainable. Spasmodic efforts are only of passing aid, but the adoption of such a movement as has been set on foot in Lancashire would prove of lasting good.

Owing to ill-health, Mr. Ruthven Pym, who is connected with so many charitable institutions, has resigned his office of Chairman and Treasurer of the **NATIONAL SOCIETY FOR THE PREVENTION OF CRUELTY TO CHILDREN.** The Society has started a new departure in the form of a children's branch, after the example of the "Band of Mercy," of the Society for the Prevention of Cruelty to Animals, which is called the Children's League of Pity. Children of well-to-do parents are asked to give five shillings annually and collect three more sums of five shillings each year towards the work of this branch, which should be sent to 7, Harpur Street, Bloomsbury.

The Bishop of London appeals for aid from those who are interested in working boys, and the improvement of boy life in London, to further an extension of work carried on at the Sea Side Camp for Boys at Deal by the London Diocesan Council for the Welfare of Young Men. It has been decided to form a brigade for Church boys in the Diocese of London on lines somewhat similar to those on which the "Boys' Brigade" was founded in Glasgow some years ago. The companies are now being enrolled under the command of Colonel Farquharson. Subscriptions will be thankfully received by the bankers, Messrs. Cocks, Biddulph, and Co., 43, Charing Cross, W.C.

An afternoon assembly on behalf of the **HOMES FOR LITTLE BOYS** at Farningham and Swanley took place on Saturday, November 28th, under the presidency of Mr. F. A. Bevan, in the Royal Albert Hall, Kensington. Among those present were Mr. W. H. Willans (Treasurer), Mr. C. R. Ford, Mr. H. Spalding, Dr. Munro Gibson, Dr. Hurlbert, U.S.A., and the Secretary, Mr. Benjamin Clarke. The Chairman said that the income last year was less than the expenditure by £2,500. There has indeed been a deficiency annually for some years past, and a debt of £5,000 has accumulated; this, by the efforts of the Committee and friends of the institution, has been reduced to £1,500. An additional income of £2,000 per annum is now needed, if the important work of the homes is to be maintained. The total income last year was £14,000; 500 lads are supported, educated, and each taught a trade. The Government Inspector speaks very highly of the way in which the boys are taught to do their work. The institution is widely and favourably known, and must by no means be allowed to dwindle for want of adequate support.

### Scraps and Gleanings.

Bingley Cottage Hospital has just received an endowment of £3,000 from Sunderland's Charity.

An election of ten boys and five girls was held at the offices of the Royal Albert Orphanage, on November 27th.

It has been agreed, in the event of larger premises being required for the Birmingham Dental Hospital, to rent these, and not to build.

The First Hospital Saturday collection in Cork has resulted in a sum of £630 being subscribed to the hospitals. This is a most encouraging result.

The Longton Cottage Hospital presents a very favourable report. Workmen's subscriptions and collections at places of worship have both increased.

The Catholic ratepayers of Clare are raising the cry that the Infirmary management is being monopolised by Protestants whilst supported by Catholic monies.

The Northern Counties Hospital for Incurables, Manchester, is making arrangements to grant out-door relief by way of pensions not to exceed £20 per annum.

In the event of the constitution of a Joint Hospital District, the Ulverston Guardians have agreed to let Oarley Infectious Hospital to the Joint District Board, at £25 per annum.

Since the Hospital Tuesday and Saturday Organisation at Manchester twenty-two years ago, the public have contributed through its agency £163,643 towards the maintenance of the local charities.

A proposal to alter the rules as now in force in relation to the election of medical officers at the Derby Royal Infirmary, was rejected, as all rules are to be revised on the completion of the new institution.

The Duchess of Montrose opened a bazaar at Glasgow, held to obtain funds to purchase a site for the Deaf and Dumb Institute. The Duchess made a speech, which was translated to the deaf and dumb inmates as she proceeded.

At the British Home for Incurables, Clapham, a sale of the patients' work, for their own benefit, was held at the institution, on the 23rd and 24th November. The sale was opened on Monday, the 23rd, by the Countess Amherst.

The Longton Cottage Hospital, Newcastle, has occupied its new and larger premises for one year, and we are glad to see that the prophesies of greatly increased expenditure has not been realised, and that workmen's subscriptions have increased.

Provision for hospital accommodation for infectious cases is now said to be ready at Orsett, at the Little Shurreck Hospital. An attendant has undertaken to nurse cases, and a Medical Officer for a remuneration of £10 a-year, will attend cases.

The Queenstown Dipensary has elected a new compounder. He is described by one of the committee at a meeting as a "gentlemanly, modest, mild man." We congratulate the Dispensary, but many more such will render Irish dispensary reports dull reading.

At the Belfast Royal Hospital 2,850 in-patients and 20,154 out-patients have been treated during the past year. The accounts show a deficit of £2,279. It is to be hoped that the bazaar held in aid of the Hospital will help to diminish the deficit to a considerable extent.

The annual report of the Aberdeen Hospital for Incurables shows a deficit on its accounts, for the second time in its history only. In both cases the circumstance has been owing to lack of special donations and legacies, a much less serious cause than absence of annual subscriptions.

The good people of Grays, in Essex, are doubtful of the venture of the proposed Cottage Hospital, and some careful attendants at a special meeting stated that three or four years were necessary to develope the scheme. This would be slow progress indeed, considering the need for a hospital.

At a meeting held on the 3rd inst., at the residence of Mr. John Aird M.P., Lord Coleridge in the chair, it was decided to establish a memorial to the late Mr. John Morgan, to take the form of an annuity, to be called the "John Morgan Annuity," which is to be given to the British Medical Benevolent Fund.

The annual report of the Female Mission to the Fallen states that during the past year 314 women have been placed in homes, 135 provided with situations, 55 restored to friends, 57 sent to hospitals, &c., while 398 have been temporarily assisted. The Committee appeal for funds to enable them to carry on the work.

Bournemouth possesses a very active friend of the temperance cause. He turns his attention especially to the consumption of alcohol in the hospitals of the town. He hopes ultimately to induce the management of these institutions to abolish the use of alcoholic beverages by the staff. The advisability of the scheme is open to question.

A concert is to be given at St. James's Hall, on December 11th, in aid of the building fund of the North London Hospital for Consumption, Hampstead. Spohr's "Last Judgment," and extracts from Handel Festival programme will be given, and the artists willl be Miss Anna Williams, Madame Marian McKenzie, Mr. Edward Lloyd, Mr. Robert Newman.

At the adjourned annual meeting of the Yorkshire Home for Incurables, it was proposed by some of the management to reject the new rules formulated at the previous meeting. This led to a discussion which tended to show that the institution had for some time been conducted in a very informal manner. A meeting is called for further consideration of the question in January.

Last week a matinée performance was given at the Princess's Theatre for the benefit of the Italian Hospital. The play selected was "Leah." On this occasion thetitle-part was taken by Mdme, Dubarry. The general cast consisted of professional actors and actresses, mostly connected with the Princess's Theatre. There was a large audience, especially in the reserved parts of the house.

Many items of interest are to be found in the volume just issued by the City Corporation on the "Population and Growth of the City of London." From this some idea of the awful ravages of the "Plague" may be gathered. In 1603, 30,561 persons died of it, in 147 parishes of London, Westminster, and Southwark; in 1625, 35,417 persons; and in 1665, the year of the "Great Plague," 68,596 persons.

### THE STUDENT OF MEDICINE.

[All communications for this Supplement should be addressed to The Editor of The Hospital, at 140, Strand, with the words, "Students' Column," written in the left hand top corner of the envelope.]

### EDITORIAL NOTES.

Lawn Tennis.—Considerable discussion recently occurred with regard to the new system of handicapping in place of the old bisque. It would appear that, as far as the production of close games is concerned, which after all is the primary object of handicapping, the old system appeared as effectual as the more modern one. Certainly there can be no question, that the old bisque had the advantage in the simplicity of its application over the new "quarter of fifteen." When anyone received a bisque, the time at which it was added to the score, was entirely at the player's own discretion. The umpire had nothing to do with it, and had not even to remind him of it. With the "quarter fifteen," however, it is different; this is a fixed and definite stroke which is scored by the umpire according to certain rules, and, as its application varies according to whether it is "received" or "owed," it is seen how confusing it becomes, both to the umpire and to the player. For instance, if a "quarter fifteen" is received, the stroke is given at the beginning of the second and every subsequent fourth game; but if it is "owed," it is scored at the beginning of the first and every fourth game. "Two quarters fifteen" are scored at the beginning of the second and every subsequent alternate game, but if "owed," they are taken at the beginning of the first and every subsequent alternate game. Also when "three quarters fifteen" are received, they come in at the beginning of the three last of every sequence of four games, but if "owed" the score is taken at the first, third, and fourth of every series of four games.

Modern Advertisements.—From a Sussex paper: "Lady having care of two little boys with ringworm, wishes to meet with one or two others to share their educational advantages." From the *Lancet*: "A lady with furniture desires to meet with medical man who would join her in taking a house for the reception of patients, and where he might also practise." "To Christian Medical Men.—Wanted, by M.D., M.S., aged thirty, late hospital resident, thoroughly experienced in all branches of general practice, a partnership with a Christian medical gentleman. Is teetotaller, non-smoker, and an anti-vivisectionist, &c." From the *Standard*: "'What lovely eyes.' Sunday in the Park, Church parade. . . Will either of the two gentlemen who twice made this remark when passing a lady dressed in white and gold, write to her? Address 'Queenie.'" . . .

### NOTES FROM THE SCHOOLS.

### University of Durham College of Medicine.

The annual students' dinner was held on Wednesday, November 18th, at the County Hotel, under the presidency of Dr. Limont; vice-chairman, Professor Bedson. There was a fair attendance, about seventy being present, among whom may be mentioned Dr. Page, Dr. McBean, Dr. Howden, and Dr. W. C. Arnison. After the usual toasts to the "Queen and Royal Family" and to the "United Services" had been duly honoured, others were proposed, bearing more immediately on the object of the gathering. Mr. Whitling proposed "The Staff of the Infirmary and Medical College," which was acknowledged by Dr. W. C. Arnison. Mr. Vissen responded on behalf of "The Students," whose health was proposed by Dr. Page. Dr. Howden followed with the toast of "The Guests," to which Mr. Fenwick replied. "The Chairman" and "Vice-Chairman" were proposed respectively by Mr. Battiscombe and Mr. Peacock. To the toast of "Students of other Schools," proposed by Mr. Smith, Mr. W. E. Jones, of the Middlesex Hospital, responded; and the list was completed by Mr. Wood with the toast of "Old Students," responded to by Dr. Green. The proceedings were diversified by songs by Dr. Limont, Messrs. Angus, Daniel, Fenwick, Jones, Wood, and Worth. The accompaniments were very kindly and ably played by Mr. Matthews. Great credit is due to the Secretaries, Messrs. Daniel and Vissen, for their successful arrangements, not forgetting the exceptionally good get-up of the programme.

### Edinburgh University.

Students' Representative Council. — Office-bearers for 1891-92: Presidents, J. G. Cattanach, Robert Munro, M.A., and W. Lyall Wilson, M.A. Secretaries, James L. Bevans and C. R. Gillies-Smith, M.A. Executive Committee.—Divinity, John Muirhead, M.A.; law, D. Milne Watson, M.A., and Maxwell

THE STUDENT OF MEDICINE—(*continued*).

Fleming, M.A.; medicine, A. A. Scot-Skirving, M.A., A. R. Wilson, M.A., D. Macaulay, and R. Hutchinson; arts, A. J. L. Laing, R. D. Melville, and R. S. Craig.

E. V. Hare and Hounds Club.—A two miles handicap was held on the 21st, at Powderhall, and resulted in G. W. Pollard (scratch) being first, J. Kennish (220) second, and R. E. Thomson (100) third.

### APPOINTMENTS.

At St. Thomas's Hospital the following appointments have been made: A. Banks, L.R.C.P., M.R.C.S., and L. G. Scudamore, L.R.C.P., M.R.C.S., clinical assistants in the skin department; A. Dalzell, L.R.C.P., M.R.C.S., clinical assistant in the throat department; C. S. Wallace, L.R.C.P., M.R.C.S., clinical assistant in the ear department; C. R. Box, B.Sc. Lond., L.R.C.P., M.R.C.S., and T. H. Kellock, M.A., M.B., B.C. Camb., F.R.C.S., L.R.C.P., assistant house surgeons; L. Cobbett, F.R.C.S., L.R.C.P., J. R. Harper, L.R.C.P., M.R.C.S., T. H. Haydon, B.A., B.B., B.C. Camb., L.R.C.P., M.R.C.S., and C. Wyman, M.A., M.B., B.C. Camb., L.R.C.P., M.R.C.S., house surgeons; J. J. Perkins, B.A. Camb., L.R.C.P., M.R.C.S., and D. F. Shearer, B.A., M.B., B.Ch. Oxon, non-resident house physicians; C. Latter, B.A., M.B., B.C. Camb., and J. J. Perkins, B.A. Camb., L.R.C.P., M.R.C.S., resident house physicians; S. G. Toller, L.R.C.P., M.R.C.S., junior, and C. H. Usher, B.A., M.B., B.C. Camb., senior ophthalmic house surgeons; W. L. Wainwright, L.R.C.P., M.R.C.S., junior, and W. F. Umney, M.B. Lond., L.R.C.P., M.R.C.S., senior obstetric house physicians. Charles A. S. Dalgleish, M.B., M.S. Durh., has been appointed house physician to the Radcliffe Infirmary, Oxford; B. W. Longhurst, M.R.C.S., L.R.C.P., has been appointed house surgeon to King's College Hospital; William Russell, M.D., C.M., F.R.C.P. Edin., has been appointed assistant physician to the Royal Infirmary, Edinburgh; G. A. Sutherland, M.A., M.B., M.R.C.P., has been appointed physician to the North London Hospital for Consumption. At the East London Hospital for Children and Dispensary for Women, Shadwell, H. J. Campbell, M.B., L.R.C.P., M.R.C.S., has been appointed assistant physician. At the Brighton Throat and Ear Hospital, Herbert Connop, B.A., Camb., L.R.C.P., has been appointed non-resident house surgeon. At Addenbrookes Hospital, Cambridge, F. Deighton, M.A., M.B., M.R.C.S., has been appointed assistant surgeon. At King's College, R. T. Hewlett, M.B., Lond., M.R.C.S., has been appointed demonstrator of Bacteriology. At Charing Cross Hospital, E. W. M. Higgs has been appointed house surgeon. At the London Hospital, Charles T. W. Hirsch, M.R.C.S., L.R.C.P., L.S.A., has been appointed senior dresser in the out-patient department. At the Royal Hospital for Diseases of the Chest, W. Kent Hughes, M.R.C.S., L.R.C.P., has been appointed house physician. At the Nottingham General Hospital, T. Alfred Waring, M.B., Lond., M.R.C.S., L.R.C.P., has been appointed resident surgical assistant. At the Sheffield Public Hospital and Dispensary, George Wilkinson, M.B., B.C., Camb., has been appointed house surgeon.

### VACANCIES.

The following vacancies are announced this week, the amount of salary in each case being given after the name of the institution:

### Resident Medical Officers.

Dudley Dispensary, Resident Medical Officer—Salary £130.

Northern Infirmary, Inverness, House Surgeon and Apothecary—Salary £100, with board, &c.

North Staffordshire Infirmary and Eye Hospital, Hartshill, Stoke-upon-Trent, House Physician—Salary £100, with board, &c.

Sunderland Infirmary, House Surgeon—Salary £80, with board, &c.

Wolverhampton Eye Infirmary, House Surgeon—Salary £60, with board, &c.

York County Hospital, Senior House Surgeon—Salary £100, with board, &c.

### Non-Resident Medical Officers.

Dental Hospital of London, Leicester Square, Dental Surgeon, and Assistant Dental Surgeon, and Assistant Anæsthetist.
Middlesex Hospital, Assistant Physician.
St. Thomas's Hospital, Demonstrator of Physiology.
Western General Dispensary, N.W., Honorary Surgeon.

